# snpAIMeR: R package for evaluating ancestry informative marker contributions in non-model population diagnostics

**DOI:** 10.1093/bioinformatics/btae377

**Published:** 2024-06-17

**Authors:** Kim L Vertacnik, Oksana V Vernygora, Julian R Dupuis

**Affiliations:** Department of Entomology, University of Kentucky, Lexington, KY 40546, United States; Department of Entomology, University of Kentucky, Lexington, KY 40546, United States; Department of Entomology, University of Kentucky, Lexington, KY 40546, United States

## Abstract

**Motivation:**

Single nucleotide polymorphism (SNP) markers are increasingly popular for population genomics and inferring ancestry for individuals of unknown origin. Because large SNP datasets are impractical for rapid and routine analysis, diagnostics rely on panels of highly informative markers. Strategies exist for selecting these markers, however, resources for efficiently evaluating their performance are limited for non-model systems.

**Results:**

snpAIMeR is a user-friendly R package that evaluates the efficacy of genomic markers for the cluster assignment of unknown individuals. It is intended to help minimize panel size and genotyping effort by determining the informativeness of candidate diagnostic markers. Provided genotype data from individuals of known origin, it uses leave-one-out cross-validation to determine population assignment rates for individual markers and marker combinations.

**Availability and implementation:**

snpAIMeR is available on CRAN (https://CRAN.R-project.org/package=snpAIMeR).

## 1 Introduction

For population diagnostics and assigning individuals to their population of origin, single nucleotide polymorphism (SNP) markers facilitate rapid panel development for a wide range of taxa. This is due to relatively accessible protocols, where SNPs can be developed from individuals of unknown parentage and genotyped with or without a reference genome (Ellegren 2014, Andrews *et al.* 2016). Despite the ease of data generation, the dense genomic coverage of modern SNP datasets is often not feasible or economical for diagnostics. For example, applied contexts like invasive species monitoring (which frequently involve non-model taxa) require rapid, high-throughput, and cost-effective sample processing for species identification and geographic source determination (Doellman *et al.* 2020, Makombu *et al.* 2022). To maximize sample processing, diagnostic panels minimize genotyping effort by using ancestry informative markers (AIMs) that have high discriminatory power (Rosenberg *et al.* 2003).

SNP AIMs are often selected by calculating and ranking a discriminatory metric for each locus; popular metrics include population genetics statistics like *F*_ST_ and informativeness measures like principal component loadings (Muñoz *et al.* 2015, Liang *et al.* 2019). Once ranked, highest-scoring loci are selected for the diagnostic panel. The panel is then validated for its ability to detect population structure in known samples, whether with multivariate ordination [e.g. principal component analysis (PCA) or discriminant analysis of principal components (DAPCs, Jombart *et al.* 2010)] or model-based estimation of ancestry [e.g. ADMIXTURE (Alexander *et al.* 2009) or STRUCTURE (Evanno *et al.* 2005)].

Although panel size depends on the diagnostic task in question and the level of population divergence in the system (Liang *et al.* 2019), candidate AIMs can be further filtered by their information content. SNPs from whole-genome datasets are expected to have a certain amount of informative redundancy due to gametic/linkage disequilibrium, therefore, directly testing the efficacy of candidate diagnostic SNPs is vital for understanding their power and limitations. Methods for non-model taxa, however, are limited. Current software programs are intended for human populations (Zhao *et al.* 2019, Pfaffelhuber *et al.* 2020), where genomic resources and data greatly exceed what is available for non-model taxa. Furthermore, the relative complexity to interpret and execute these tools represents a significant barrier for non-specialist users. Finally, they are intended for forensic genetics applications which necessitates a high degree of accuracy; in urgent phytosanitary or conservation interventions, a heuristic marker search may suffice to prioritize rapid panel deployment (Dupuis *et al.* 2019, Janjua *et al.* 2020).

Here, we present snpAIMeR, an R package that evaluates marker contribution for effective population diagnostics. It is intended as an additional filtering step for candidate AIMs by evaluating marker informativeness and addresses a need for AIM software that is relatively accessible to non-specialist users. Provided candidate AIMs and genotype data from individuals of known origin, snpAIMeR assesses the ability of individual markers and marker combinations to correctly assign individuals to their population of origin. In doing so it can help minimize population diagnostic panel sizes and facilitate panel development for non-model taxa.

## 2 Design and usage

snpAIMeR is based on the adegenet population genetics R package (Jombart 2008). The user provides a STRUCTURE (Evanno *et al.* 2005) formatted file (.str or .stru) with candidate SNPs and individual population assignments, a range of panel sizes to test, a specified threshold value for successful test data assignment, and a specified number of cross-validation replicates. Then, using the provided markers and panel sizes, snpAIMeR creates all possible marker combinations and tests each one with leave-one-out cross-validation. For each cross-validation replicate, each population is randomly split into two groups for training (75% of individuals) and testing (25% of individuals). The training dataset is subject to a discriminant analysis of principal components (DAPC) (Jombart *et al.* 2010) which is used to assign source populations to the test individuals; assignments are confirmed with the input data. DAPC is a multivariate discriminate approach that is computationally inexpensive and, as a classification method, has been shown to be both accurate and powerful at delimiting hierarchical relationships (Kanno *et al.* 2011). For individual markers and marker combinations, the rate of correct population assignment is the average of all cross-validation assignment rates. For panel sizes, the assignment rate is the average assignment rate of all possible marker combinations for that size.

While alternative classifiers such as Bayesian approaches (STRUCTURE, Evanno *et al.* 2005) or machine learning methods (Chen *et al.* 2018) are popular and commonly used in population genetics, their computational requirements make them burdensome for the general approach of snpAIMeR, which tests all possible marker combinations, thus leading to thousands of individual analyses that are each replicated hundreds to thousands of times. In preliminary analyses comparing snpAIMeR to the machine learning approach of assignPop (Chen *et al.* 2018), we found the latter takes upwards of 10 times longer to test panel sizes and marker combinations in the same way as snpAIMeR (Supplementary Table S1).

## 3 Implementation

**Figure 1.**
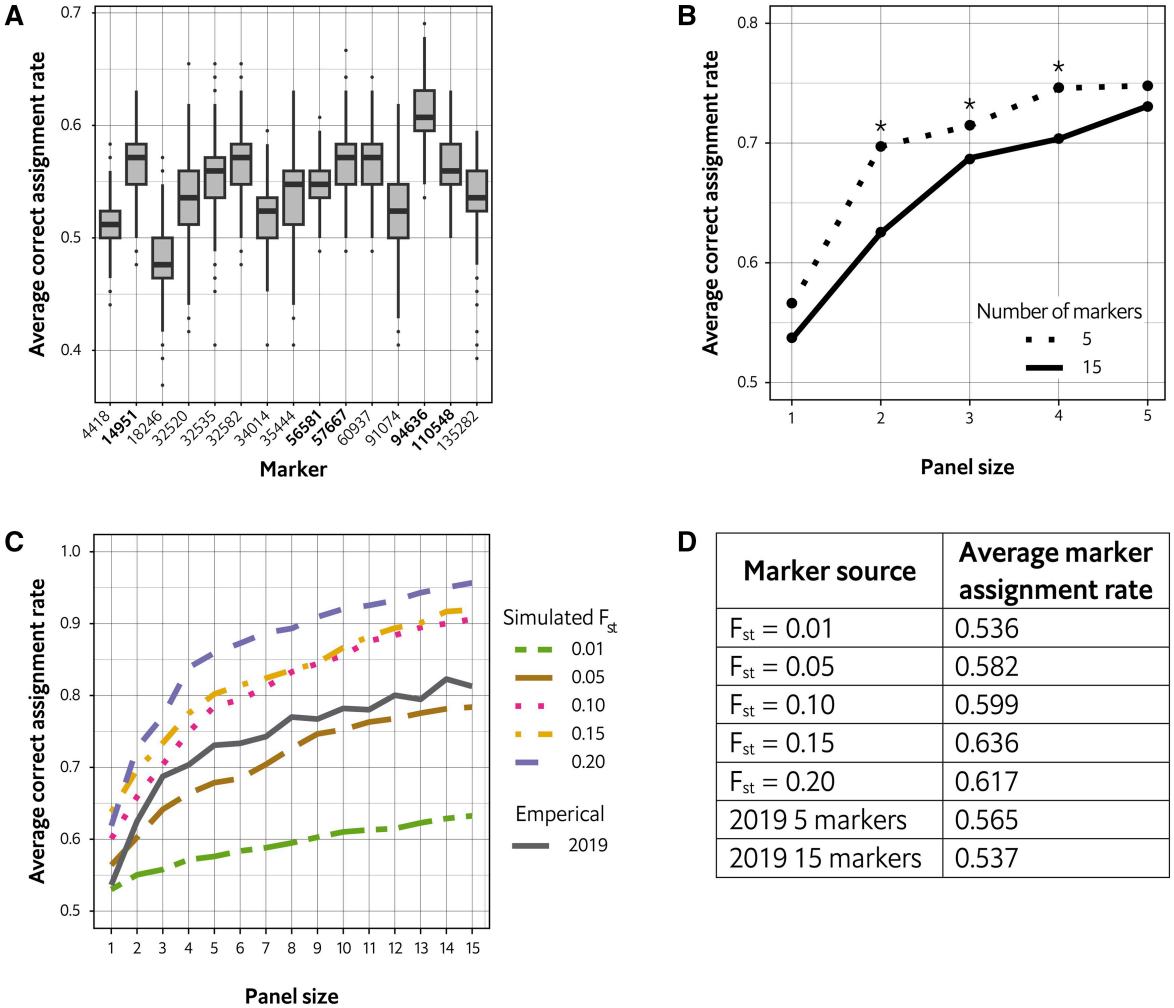
Population diagnostic marker selection using snpAIMeR. (A) Assignment rate of 15 markers selected from Dupuis *et al.* (2019). Five markers were selected for a hypothetical diagnostic panel (marker names in bold). (B) Comparison of panel size assignment rates for the 15- and 5-marker groups. * indicates assignment rates that differ significantly at *P* < 0.05 (Student’s *t*-test). (C) Comparison of panel size assignment rates for simulated *F*_ST_ populations. Dotted lines represent simulated populations, the solid line indicates the 15-marker group (*F*_ST_ = 0.311). (D) Average marker assignment rate for all datasets.

To demonstrate diagnostic marker selection using snpAIMeR, we tested empirical data from four wild subpopulations (34, 57, 86, and 159 individuals) of *Anastrepha ludens*, a pestiferous fruit fly (Dupuis *et al.* 2019). This data represents 28 SNPs (selected from a restriction-site associated DNA sequencing dataset of 2081 SNPs), from which 15 were selected based on *F*_ST_ and DAPC loading values. First, the rate of correct population assignment was determined for each of the 15 markers (Fig. 1A), from which the top five were selected. Second, both the 5- and 15-marker groups were evaluated for panel sizes of 1–5. In three of the five panel sizes, the 5-marker group had a significantly greater assignment rate than the 15-marker group (Fig. 1B), demonstrating that similar if not better results can be achieved by selecting a subset of the most informative markers.

To assess how population differentiation affects marker diagnostic power, we chose a range of *F*_ST_ values (0.01, 0.05, 0.10, 0.15, 0.20) and for each, simulated 20 populations using the FILEST R package (Chaichoompu 2021). Each simulated population had 2000 individuals divided into two subpopulations of 1000 and was represented by 15 markers. The simulated populations were evaluated for panel sizes one through 15, and as expected, markers from less differentiated populations had more limited diagnostic power (Fig. 1C). However, we also evaluated the *A. ludens* 15-marker group, and although it had a mean *F*_ST_ of 0.311, these markers had moderate diagnostic power compared to the simulated datasets (Fig. 1C, D). This may indicate that empirical *F*_ST_ values have limitations when predicting marker diagnostic power, perhaps due to missing data, the number of subpopulations, or the number of individuals in each subpopulation.

## Supplementary Material

btae377_Supplementary_Data

## Data Availability

The data underlying this article will be shared on reasonable request to the corresponding author.
