# Supplementary material for: snpAIMeR: R package for evaluating ancestry informative marker contributions in non-model population diagnostics
Source: Bioinformatics. 2024 Jun 17;40(6):btae377. doi: 10.1093/bioinformatics/btae377 (PMC11194479; doi:10.1093/bioinformatics/btae377)
Supplement: btae377_Supplementary_Data [file btae377_supplementary_data.pdf]

Panel size	Number of possible combinations (out of 15 markers)	assignPOP runtime for a single combination (seconds)	assignPOP estimated total runtime for all combinations (seconds)	snpAIMeR runtime for a single combination (seconds)	snpAIMeR estimated total runtime for all combinations (seconds)
1	15	NA	NA	0.395	5.927
2	105	4.627	485.929	0.389	40.817
3	455	4.690	2,134.199	0.413	187.757
4	1,365	5.124	6,994.723	0.450	614.067
5	3,003	5.087	15,278.376	0.450	1,350.572
6	5,005	5.171	25,881.601	0.477	2,385.654
7	6,435	5.224	33,618.724	0.525	3,381.212
8	6,435	5.085	32,723.950	0.502	3,233.387
9	5,005	5.195	26,003.207	0.582	2,912.540
10	3,003	5.368	16,122.516	0.543	1,631.707
11	1,365	5.383	7,347.626	0.570	777.638
12	455	5.423	2,467.364	0.652	296.772
13	105	5.435	570.651	0.697	73.215
14	15	5.723	85.847	0.621	9.310
15	1	5.524	5.524	2.002	2.002
Sum	*32,767*	*68.432*	*169,720.237*	*9.267*	*16,902.577*

Table S1. Runtimes for testing the assignment rate accuracy of combinations of 15 candidate *A. ludens* diagnostic markers. assignPOP (Chen et al. 2018) uses machine learning classifiers to develop predictive models (command used: assign.MC(data, train.inds=c(0.75), train.loci=c(1), loci.sample="fst", iterations=1000, model="randomForest", dir=output_folder)). snpAIMeR (this paper) uses discriminant analysis of principal components (DAPC, Jombart et al. 2010) as a classifier for leave-one-out cross-validation with 75% of samples used for training and 25% for testing (settings used: 1000 replicates). For a given program and panel size, we determined (1) the total number of possible combinations (out of 15 markers) and (2) obtained the runtime for a single combination. To estimate the total runtime for all combinations within a panel size, we multiplied (1) by (2). In snpAIMeR, the first combination runtime is longer than that for subsequent combinations, so we recorded the runtime for the second combination (except for panel size 15). The estimated run time for all 15 panel sizes was 47 hours for assignPOP and five hours for snpAIMeR. All analyses were performed on a macOS 14.4.1 system with 14 processing cores and 36 GB RAM.
